# Trends in national-level school feeding policy objectives worldwide: Japan and multiple countries

**DOI:** 10.1186/s41182-025-00818-1

**Published:** 2025-11-24

**Authors:** Takeshi Akiyama, Sachi Tomokawa, Mika Kigawa, Fumiko Shibuya, Mami Hitachi, Yuko Teshima, Luna Shimabukuro, Tomoko Hato, Aiko Inoue, Akira Kurishima, Eri Mochimaru, Juri Murata, Noriko Saito, Sachi Tensho, Kenzo Takahashi, Jun Kobayashi

**Affiliations:** 1 Faculty of Nursing, Nagano College of Nursing, 1694 Akaho, Komagane City, Nagano, 399-4117 Japan; 2 The Japanese Consortium for Global School Health Research, 1076 Kiyuna, Ginowan, Okinawa 901-2720 Japan; 3 Faculty of Education, Shinshu University, 6-Ro, Nishi-Nagano, Nagano City, Nagano, 380-8544 Japan; 4 Faculty of Health and Social Services, Kanagawa University of Human Services, 1-10-1 Heiseicho, Yokosuka City, Kanagawa, 238-8522 Japan; 5 Faculty of Nursing, Yamagata Prefectural University of Health Sciences, 260 Kamiyanagi, Yamagata City, Yamagata, 990-2212 Japan; 6 Department of Epidemiology, Institute of Tropical Medicine, Nagasaki University, 1-12-4 Sakamoto, Nagasaki Nagasaki City, Nagasaki, 852-8523 Japan; 7 Department of Community and Global Health, Graduate School of Medicine, the University of Tokyo, 7-3-1 Hongo, Bunkyo-ku, Tokyo, 113-0033 Japan; 8 Department of Global Health, Graduate School of Health Sciences, University of the Ryukyus, 1076 Kiyuna, Ginowan, Okinawa 901-2720 Japan; 9 Graduate School of Public Health, Teikyo University, 2-11-1 Kaga, Itabashi-ku, Tokyo, 173-8605 Japan; 10 Division of Rheumatology, Department of Internal Medicine, Tokyo Women’s Medical University. School of Medicine, 8-1 Kawada-cho, Shinjuku-ku, Tokyo, 162-8666 Japan; 11 Tetsuikai Research Institute, Tokyo, Japan; 12 Department of Radiation Health Management, Fukushima Medical University School of Medicine, 1 Hikarigaoka, Fukushima City, Fukushima, 960-1295 Japan

**Keywords:** School feeding, School feeding policy, School health, School meal

## Abstract

Public policy denotes a government’s formal intent to address specific issues in pursuit of defined objectives. The vision and mission of school feeding have progressively expanded. This study examined school feeding policy objectives across multiple countries, drawing on data from the Global Survey of School Meal Programs, and identified 23 relevant policies. The objectives were classified into five domains: health and nutrition, education, social protection, agriculture, and other areas. Japan is particularly notable for distinctive aims, including enhancing school life, fostering sociability and cooperation, promoting appreciation of nature, and cultivating respect for life and the natural world.

**Supplementary Information:**

The online version contains supplementary material available at 10.1186/s41182-025-00818-1.

Public policy is a statement of what a government intends to do about a problem to achieve specific goals. Policies allow us to understand the declaration of intentions, wishes, principles, or expression of desires of the country. The policy would be law, regulation, procedure, administrative action, incentive, or voluntary practice of governments. Public health policy fosters system development, organizational change, and influences individual behavior to improve and promote health. The structural and policy (macro-level) factors form the population’s health behavior, interdependent with the community/cultural influences (meso-level), and individual/behavioral factors (micro-level) [1].

School feeding programs, often referred to as school meal programs, are interventions that provide meals regularly to schoolchildren. The form of school feeding is not uniform worldwide. In the Global Child Nutrition Survey, the most common school food delivery is in-school meals (82.1%) across 184 school feeding programs, followed by take-home rations (39.1%), in-school snacks (29.3%), and other [2]. Among the in-school meals, 89% was lunch, 40% was breakfast, and 11% was dinner, in settings such as boarding houses [3]. The frequency among programs was mostly 5 days a week (70.5%), followed by a small number of seven (3.8%), six (3.3%), and four (3.3%) times a week [4]. Often, school feeding programs select areas, schools, grades, or students with poverty or other disadvantages [2]. According to 2024, the Global Child Nutrition Foundation (GCNF) survey data, 27.1% of school feeding programs were estimated to be universal provision [4]. The provision might be only for those who prefer to eat in the school canteen instead of going home for lunch, such as in France, while Japanese students generally have uniform school lunch in their classrooms during all the school days as an educational component. Majority of the school meal programs were estimated with some type of family contribution, such as in-kind contributions on a voluntary basis, providing firewood, staple foods, or condiments [5].

Today, the objectives of school feeding are considered to benefit health/nutrition, education, social protection, and agriculture [6]. Traditionally, school feeding was considered a way to deliver food aid to vulnerable children. Then, countries, development agencies, and policymakers viewed multiple returns from school feeding. Early on, the launch of the FRESH framework (Focusing Resources on Effective School Health) promoted school feeding as improving educational quality and equity with the school feeding’s benefit to enrollment and attendance [6]. School feeding was implemented with the object of social protection, such as South Africa’s Care and Support for Teaching and Learning Program in 1991 [7] and India’s national mid-day meal program [8]. Now, school feeding is recognized as among the most extensive and effective safety nets in the world [9].

According to The 2021 Global Survey of School Meal Programs, the objectives of school feeding programs are indicated as follows: “To meet nutritional and health goals”(93.5%), “to provide a social safety net” (72.8%), “to meet education goals”(84.2%), “to prevent or mitigate obesity”(34.8%), “to meet agricultural goals” (40.1%), and “other” (18.5%). “Other” included responses such as reducing food security, increasing national ownership, and enhancing parent participation [2]. Today, the vision and mission of school feeding is expanded, responding to financial, food crisis, and COVID-19 pandemic [9, 10]. Universal school meals are emerging as the most popular program approach, which is ensuring equity and cost effectiveness of scale [11]. The current urgent issues such as climate change, social, and economic disparities have gained increasing prominence [12].

As there is a few policy-level information, we analyzed the objectives of school feeding policy using the data from the 2021 and 2024 Global Survey of School Meal Programs (Table 1)^1^^,^ 2. The methodology is summarized in Appendix 1. The objectives were widely distributed across those categories: health and nutrition, education, social protection, agriculture, and others. Then all countries covered “Health and nutrition” as the objective^3^. For health and nutrition, the role of school feeding would become more important as current concerns about obesity intensify the focus on school environments and the standard of school meals. Furthermore, educational benefits have been firmly established in the countries’ policies. There are countries where securing enrollment is explicitly mentioned as an objective. In India, the focus is on children from poor households; in São Tomé and Príncipe, on children facing gender-related, mental, social, or physical conditions; and in the Republic of Congo, on disadvantaged groups or those with special needs, and indigenous populations. In each case, school feeding is positioned as a measure to address the specific groups that require particular attention in the national context.

**Table 1:** Objectives of the school feeding policy across countries

Country	Name of the law, policy, or standard (the language of the name is from the data source)	Health and nutrition	Education	Social protection (household food security)	Agriculture (rural economy, food system)	Other1	Other2
Angola	Decreto presidencial nº 138/13 de 24 de Setembro	✓	✓				
Bangladesh	National Education Policy 2010		✓				
Brazil	LEI Nº 11.947, DE 16 DE JUNHO DE 2009	✓	✓				
Cabo Verde	Lei nº 89/VIII/2015 que estabelece o regime jurídico de alimentação e saúde escolar	✓	✓				
Ecuador	Ley Orgánica de Alimentación Escolar	✓					
Ghana	National School feeding Policy	✓		✓	✓	Strengthen collaboration and coordination between national and sub-national actors in implementing school feeding	
Guatemala	1) Ley de Alimentación Escolar, Decreto 16–2017 2) Reglamento de la Ley de Alimentación Escolar, Acuerdo Gubernativo 183–2018 (Only cited to the Agriculture)	✓	✓		✓		
Honduras	Ley de Alimentación Escolar mediante decreto N° 125–2016	✓	✓				
India	The National Programme of Nutritional Support to Primary Education (NP-NSPE)	✓	✓	✓			
Jamaica	National School Nutrition Policy (DRAFT)	✓	✓	✓	✓	Facilitating improvements in the management and monitoring of the School Feeding Programme (SFP)	
Japan	School Lunch Act Fundamental Law of Nutrition Education	✓	✓				
Kenya	National school meals and implementation strategy	✓	✓		✓	Strategic Objective 4. To develop and implement a sustainable national school meals and nutrition programme	Strategic Objective 5. To promote partnerships and multi-sectoral coordination for complementary support and effective implementation of the school meals and nutrition programme
Namibia	Namibia School Feeding Policy	✓	✓		✓		Strengthen coordination and sectoral linkages in the management, implementation and monitoring of the Namibia School Feeding Programme
Nepal	Joint Action Plan 2071/72–2076/77 School Health and Nutrition	✓	✓			To incorporate SHN (School Health and Nutrition) program in SIP *School Improvement Plan)/annual work plan of schools”	
Panama	Ley 115 de 5 de diciembre de 2019	✓	✓	✓			
Philippines	Republic Act 11,037, Masustasyang Pagkain para sa Batang Pilipino Act	✓					
Republic of Congo	Politique Nationale d’Alimentation Scolaire	✓	✓	✓	✓	Strategic axis 5: Trade and Industry: The PNAS should lead to: (i) enhancing food value chains, production, processing, fortification, preservation and marketing of local products; (ii) creating job opportunities in these chains; (iii) ensuring a stable income for small producers; (iv) increasing food quality and safety	The long-term objective of the Policy is to support the government’s actions for the “development of human capital with the strengthening of the education system and the improvement of the health system … with a view to improving the development of other sectors The specific objectives of the present policy, defined during the national consultation on school nutrition, are translated into strategic axes reflecting the interrelation between school nutrition and the various sectors of economic and social development
Saint Kitts and Nevis	St. Kitts and Nevis Nutrition and Food Security Policy	✓		✓	✓	(2.3.4Food Utilization)Policy Statement 3: ・Developing national guidelines for the preparation and sale to children of school meals that promote health and wellness	
Sao Tome and Principe	Lei n.⁰ 1/2023	✓	✓	✓	✓		
Sierra Leone	2024 national school feeding policy	✓	✓	✓	✓	The long-term objective of this policy is to ensure that school feeding contributes to the vision, mission, and goals of the GoSL as articulated in the National Development Plan and the Education Sector Plan, by providing locally produced and processed nutritious and healthy school meals to all pupils enrolled in pre-primary and basic and junior secondary education schools, contributing to the improvement of children’s nutritional status. GoSL: Government of Sierra Leone	
Spain	Orden del Ministerio de Educación y Cultura de 1992,	✓	✓				
United States of America	National School Lunch Act 1946	✓		✓	✓		
Zambia	National HGSM Strategy	✓	✓		✓.;		

As for the benefit of social protection, nine countries mentioned in their policy, and it was supported by the fact that school meals are considered one of the most widespread and significant interventions [13]. A few countries covered agricultural benefits. However, currently, FAO and WFP promote obtaining their supplies from local smallholder farmers, which is known as “home-grown school feeding (HGSF)”. The other objectives we found were regarding strengthening coordination or social/economic development. Since children’s health, education, agriculture, and social protection are all closely tied to social and economic development, the use of this expression is reasonable when considered within the recent expanding contexts of school feeding. Furthermore, the result seems to have a similar trend with a study conducted on the school feeding program using the GCNF dataset, the relevance of nutritional objectives in programs as income level decreases [13, 14]. This tendency can be considered to reflect the greater benefit to the investment and wider expectations placed on school feeding in resource-constrained countries.

On the other hand, examining the context of the objectives reflected in the policies in detail, Japan stands out in terms of educational benefit from school feeding. It covers (i) To maintain and promote health through proper nutrition, the remaining objectives reflect distinctive intentions; (ii) to deepen students’ correct understanding of food in daily life, to cultivate the ability to make sound dietary decisions, and to nurture desirable eating habits; (iii) to enrich school life and foster pleasant sociability and a cooperative spirit; (iv) to deepen students’ understanding that dietary life is based on the benefits of nature and to cultivate a spirit of respect for life and nature, as well as an attitude that contributes to the preservation of the environment; (v) to deepen the understanding that dietary life is supported by the various activities of people involved in food and to cultivate an attitude of respect for hard work; (vi) to deepen understanding of the excellent traditional food culture of Japan and other regions; and (vii) to lead students to a correct understanding of food production, distribution, and consumption.

As shown in the policy, school lunch in Japan is considered an important means of educating children from the outset, called “lively learning materials” [15]. Initially in 1954, the School Lunch Act was enacted, and thereafter, the implementation of the school lunch program was organized in the whole county [16]. With this Act, school lunches have become not just for providing nutrition to school children. They are also an important educational means in schools [17]. The revision of the Course Study in 1958 defined school lunches as part of “School Events” among the four components of the school curriculum.

Japanese school feeding is essentially a school lunch program providing a meal with a main dish and side dishes every school day. In classrooms during all school days all over Japan, school lunch education has provided activities such as nutritional instruction through menus handed out monthly by the classroom teacher and lunchtime instruction on table manners to weed out picky eating [17]. Children carried out serving and cleanup duties. The students learn the practice of role-sharing and self-control with rotating rosters of preparation [17]. However, recently there are concerns about whether the Japanese approach, such as a uniform menu and involvement, is appropriate, given issues including food allergies, obesity, school refusal, and diverse family backgrounds [18].

The beginning of Japan’s school lunch system was not separable from the social situation of the era. After the interruption due to World War II, school lunches started with supplies: Licensed Agencies for Relief in Asia (LARA) from the United States (US). The US side aimed to stabilize social tension in occupied Japan by addressing food shortages and improving public sentiment. On the other hand, Japanese educators and policymakers embraced the idea of uniformly providing school lunches, viewing it as eliminating the sense of inequality and fostering democratic values [16]. Bread-based school lunches with imported flour from the US were introduced, which were different from Japanese food culture, but it is scoping a new market for the US flour industry [16].

Japanese school feeding policy emerged in response to the strong commitment to popular nation-building and extremely severe food shortages, and its food supply and background were further shaped by the political and economic circumstances of the postwar period. Policies often may face tensions undermined by conflicts of interest. Later, the Japanese government shifted to rice-based school lunches to promote traditional dietary practices and to absorb surplus rice [16]. Similarly, in the United States, ‘government cheese’ served a similar role for dairy products; and in the European Community, the School Milk Scheme was launched in 1977 both to support child nutrition and to manage surplus milk [19]. These examples show how school feeding has often combined nutrition policy with agricultural policy.

Nevertheless, we consider that the educational dimension of Japan’s school feeding program may provide useful insights for future discussions. At present, food systems education is an increasingly important component of school feeding programs, as holistic food education should be institutionalized within national school systems, designed with an action-oriented focus, implemented regularly, and made available to all grades [12]. Prioritizing real-life and practical activities, such as raising waste awareness, has been recommended [12]. The long-standing practices of Japanese school feeding can serve as a reference for many countries in expanding their policies. But it should also be noted that historical and cultural factors may play a highly significant role. For example, while participation in school meals in France is left to individual choice, in Japan the program has been institutionalized as part of the curriculum with universal participation. Such social, economic, and cultural aspects should be considered in future analyses.

## Supplementary Information

Additional file 1 (Title of data: Appendix 1. Method, Description of data, The file is supplemental detailed information about 1) the methodology to identify policy the countries which participated in The Global Child Nutrition Foundation (GCNF) survey from its data set and 2) The list of 23 countries and the information reference for the school feeding policy.)

Additional file 2 (Title of data: Appendix 1. The countries which participated and did not participate in The Global Child Nutrition Foundation (GCNF) survey 2021. Description of data. The file is supplemental detailed information about the countries which participated and did not participate in The Global Child Nutrition Foundation (GCNF) survey 2021from its data set.)

Additional file 3 (Title of data: Appendix 1. Objectives of the school feeding policy across countries. Description of data. The file is supplemental detailed information and description in the school feeding policy of the countries involved in this study.)

## Data Availability

The data are available with the additional file.

## Abbreviations

FAO: Food and Agriculture Organization of the United Nations
FRESH: Focusing Resources on Effective School Health
WFP: World Food Programme

## Footnotes

1 The information sources and documents were obtained through web search; then the documentation was limited to the ones that responded to survey and were archived on the internet.

2 The GCNF survey relied on the cooperation of each country, and there are countries which did not participate (Appendix 2). Among those countries were some of the poorest as well as some developed countries, raising concerns about potential response bias. The analysis of the present study based on only 23 countries among 98 countries responded to the policy name. Among them, six countries have been excluded from the analysis due to the eligible languages, and the researchers could not identify the other 69 countries in the web search.

3 Attention should be paid when analyzing policy; it is important to distinguish between the enactment of a policy and its subsequent consolidation. World Bank developed Systems Approach for Better Education Results (SABER) conducted a survey which evaluated the progress toward achieving the policy goals among the countries [20]. The results indicated that even though school feeding policy framework is comparatively well established, the countries need support to strengthen program design and implementation, and financial capacity [20]. Further, in some countries, school meals might be administered by local governments, such as Belgium. Adding further complexity, in France, there is no law specifically on school meals; but school meal is provided and there are regulations covering conditions of provision, including non-discrimination, pricing, and ingredients. In addition, the *Stratégie de prévention et de lutte contre la pauvreté* includes school breakfasts for children from low-income households as part of efforts to ensure their fundamental rights.
