# Supplementary material for: Trends in national-level school feeding policy objectives worldwide: Japan and multiple countries
Source: Trop Med Health. 2025 Nov 24;53:172. doi: 10.1186/s41182-025-00818-1 (PMC12642336; doi:10.1186/s41182-025-00818-1)
Supplement: Supplementary file 1 — Additional file 1 (Title of data: Appendix 1. Method, Description of data, The file is supplemental detailed information about 1) the methodology to identify policy the countries which participated in The Global Child Nutrition Foundation (GCNF) survey from its data set and 2) The list of 23 countries and the information reference for the school feeding policy.) [file 41182_2025_818_MOESM1_ESM.docx]

**Methods**

1. Source of the title of the policy in the countries.

The present study used the data from the 2021 and 2024 Global Survey of School Meal Programs [3,4]. The 2021 survey was conducted from July 2022 to March 2022, collecting data on the school year which began in 2020 from the governments. The survey targeted 194 countries to ask for the questionnaire, which was available in PDF, Word, and online survey format. Of the 194 countries, the survey received responses from 140 countries. In the survey answer format, there was a question on the existence of national policy: “Are there national laws, policies, or standards related to school feeding?” (Section B: National context II B1). Name of national law/policy/standard (2)-national school feeding policy. Of the total, 98 countries, typically ministry of education, responded by inputting the name of the policy. The present study collected the names of national school feeding policies from this section for use in the data collection. The 2024 survey was conducted by the same manner in 2024, involving 142 countries.

2 Data collection

The names of the policies in the dataset were web-searched during December 2023. The web-search results were examined and confirmed by the members of the researchers as follows: Policy may be a law, regulation, procedure, administrative, action, incentive, or voluntary practice of governments. The policy names inputted with the language, including English, Spanish, French, and Portuguese, were used for the web search of the official documents by the researcher with expertise in each language. Only official and original policy documents on the government sites or documents stored in United Nations-related organizations were downloaded for the context analysis of the objectives. The documents unrelated to school feeding policy, such as food and beverage standards, were excluded from the analysis.

Of the total 98 policies in the data set of the 2021 survey, the policies in other languages except English, Spanish, French, and Portuguese, namely German (Austria), Finnish, Tajik, Slovak, Hungarian, and Italian, were excluded from the web search results. Of the remaining 93 policies, only 22 countries’ policies were identified and involved in the analysis. The other documents were not found. The researchers of the present study consisted of Japanese members and therefore added the Japanese policy and its objectives, as Japanese authority did not participate in the survey. Therefore, in total, 23 countries were involved in the analysis. In 2025, the 2024 Global Survey of School Meal Programs survey data set was published involving 142 countries [4]. Also in the 2024 dataset, 98 countries response on school feeding policy and the name of the policy responded was re-checked with 2021 survey data during April 2025. Through this process, only Sao Tome Principe and Sierra Leone were updated which was already included above-mentioned 23 countries. We analyze the objective of each policy, including the following aspects: health and nutrition, education, social protection, agriculture, and others. Further, the retrieved URL at the day of the data collection is collected. The vision, mission and objectives were translated into English if the original document is not in English language by the researchers.

**Refference for Table 1.**

Angola. Decreto presidencial nº 138/13 de 24 de Setembro https://faolex.fao.org/docs/pdf/ang130771.pdf (2013) Accessed 20 Dec 2024.

Bangladesh National Education Policy 2010 http://file-chittagong.portal.gov.bd/files/www.lakshmipur.gov.bd/files/f97d6b95_2046_11e7_8f57_286ed488c766/National%20Education%20Policy-English%20corrected%20_2_.pdf (2010) Accessed 10 Dec 2024.

Brazil LEI Nº 11.947, DE 16 DE JUNHO DE 2009. https://www.planalto.gov.br/ccivil_03/_ato2007-2010/2009/lei/l11947.htm (2009) Accessed 11Dec 2034.

Cabo Verde Lei nº 89/VIII/2015 que estabelece o regime jurídico de alimentação e saúde escolar

https://faolex.fao.org/docs/pdf/cvi148344.pdf (2015) Accessed 28 Dec 2024.

Ecuador Ley Orgánica de Alimentación Escolar https://www.fao.org/faolex/results/details/es/c/LEX-FAOC101460/ (2011) Accessed 20 Feb 2025.

Ghana National School feeding Policy https://www.mogcsp.gov.gh/mdocs-posts/national-school-feeding-policy/ (2015) Accessed 20 Feb 2025.

Guatemala Ley de Alimentación Escolar, Decreto 16-2017 https://siteal.iiep.unesco.org/sites/default/files/sit_accion_files/10036.pdf (2017) Accessed 10 Dec 2023.

Guatemala Reglamento de la Ley de Alimentación Escolar, Acuerdo Gubernativo 183-2018 https://faolex.fao.org/docs/pdf/gua196828.pdf (2018) Accessed 20 Dec 2023.

Honduras Ley de Alimentación Escolar mediante decreto N° 125-2016 https://www.fao.org/faolex/results/details/es/c/LEX-FAOC174256/ (2016) Accessed 20 Feb 2025.

India The National Programme of Nutritional Support to Primary Education (NP-NSPE) https://pmposhan.education.gov.in/Files/Guidelines/10.FINAL_Guidelines_MDM_19_sept.pdf (2006) Accessed 20 Dec 2023.

Jamaica National School Nutrition Policy (DRAFT) https://moey.gov.jm/wp-content/uploads/2022/05/DRAFT-POLICY-National-School-Nutrition-Policy-.pdf (2022) Accessed 20 Feb 2025.

Japan School Lunch Act https://laws.e-gov.go.jp/law/329AC0000000160 (1954) Accessed 20 Feb 2025.

Japan Fundamental Law of Nutrition Education https://laws.e-gov.go.jp/law/417AC1000000063 (2016 ) Accessed 20 Feb 2025.

Kenya National school meals and implementation strategy https://docs.wfp.org/api/documents/WFP-0000116843/download/ (2017) Accessed 20 Feb 2025.

Namibia Namibia School Feeding Policy https://www.fao.org/docs/devschoolfoodlibraries/materials-from-countries/namibian-school-feeding-policy-(2018-2023).pdf?sfvrsn=790f88f4_5 (2018) Accessed 20 Feb 2025.

Nepal　 Joint Action Plan 2071/72 – 2076/77 School Health and Nutrition https://faolex.fao.org/docs/pdf/nep191267.pdf (2014) Accessed 20 Feb 2025.

Panama　 Ley 115 de 5 de diciembre de 2019 https://siteal.iiep.unesco.org/sites/default/files/sit_accion_files/11227.pdf (2019) Accessed 20 Feb 2025.

Philippines Republic Act 11037, Masustasyang Pagkain para sa Batang Pilipino Act　 https://web.senate.gov.ph/republic_acts/ra%2011037.pdf (2017) Accessed 20 Dec 2024.

Republic of Congo Politique Nationale d'Alimentation Scolaire https://docs.wfp.org/api/documents/WFP-0000117050/download/ (2016) Accessed 20 Feb 2025.

Saint Kitts and Nevis St.Kitts and Nevis Nutrition and Food Security Policy https://faolex.fao.org/docs/pdf/stk148759.pdf (2025) (Accessed 28 Aug 2025)

Sao Tome and Principe Lei n.⁰ 1/2023 https://faolex.fao.org/docs/pdf/sao215703.pdf (2023) Accessed 20 Apl 2025.

Sierra Leone national school feeding policy

https://mbsse.gov.sl/wp-content/uploads/2021/05/National-School-Feeding-Policy_May2021.pdf (2021) Accessed 20 Apl 2025.

Spain Orden del Ministerio de Educación y Cultura de 1992, https://www.boe.es/buscar/doc.php?id=BOE-A-1992-27221 (1992) Accessed 20 Feb 2023.

United States of America National School Lunch Act 1946 https://www.govinfo.gov/content/pkg/COMPS-10333/pdf/COMPS-10333.pdf (1946) Accessed 20 Feb 2025.

Zambia National HGSM (Home Grown School Meals (HGSM)) Strategy https://www.edu.gov.zm/wp-content/uploads/2023/02/NATIONAL-STRATEGY-ON-HGSM-2020-2024-web-003.pdf (2020) Accessed 20 Feb 2025.
